# Velamentous and Furcate Cord Insertion with Placenta Accreta in an IVF Pregnancy with Unicornuate Uterus

**DOI:** 10.1155/2013/539379

**Published:** 2013-12-23

**Authors:** Mehmet Tunç Canda, Namık Demir, Latife Doganay

**Affiliations:** ^1^Obstetrics and Gynecology Unit, Kent Hospital, 8229/1 Sok. No. 56, Cigli, 35580 Izmir, Turkey; ^2^Pathology Unit, Kent Hospital, 8229/1 Sok. No. 56, Cigli, 35580 Izmir, Turkey

## Abstract

Velamentous and furcate cord insertion with concomitant placenta accreta is a very rare and life-threatening event of pregnancy for both the mother and the fetus. Obstetricians should be cautious about umbilical cord insertion and placental adherence abnormalities in pregnancies conceived by assisted reproductive technologies (ART) particularly in women with Müllerian anomalies.

## 1. Introduction

Velamentous cord insertion is the insertion of the umbilical cord into the membranes of the placenta before reaching the placental margin and it occurs in 1.5% of term singleton placentas; however in ART pregnancies the incidence rises to 3.65% [1]. Velamentous cord insertion is associated with preterm labor-delivery, low birth weight, fetal growth restriction, abnormal intrapartum fetal heart rate patterns, low APGAR scores at 1 and 5 minutes, neonatal deaths and placental abruption [2, 3]. Furcate umbilical cord insertion is the separation of umbilical vessels prior to their attachment into the placenta. It is a very rare entity with the risk of intrapartum hemorrhage [4]. Placenta accreta is the invasion of the decidual surface of the myometrium with placental villi. In a recent series, the incidence of placenta accreta with increta and percreta was reported to be 1.7 per 10000 pregnancies and 4 per 10000 among IVF pregnancies [5]. Abnormal placentation is associated with major pregnancy complications. Unicornuate uterus is formed by normal differentiation of one mullerian duct and either partial or complete failure of the differentiation of the other duct with an incidence of one in 4020 women in the general population and among infertile women 0.4% [6, 7]. Unicornuate uterus is associated with reduced fertility, miscarriage, and preterm delivery [7].

Herein, we report for the first time a very rare association of velamentous and furcate cord insertion with placenta accreta in a pregnancy achieved by in vitro fertilization (IVF) in an infertile patient with unicornuate uterus.

## 2. Case Presentation

A 39-year-old, primary infertile woman achieved a pregnancy on the 4th IVF attempt with the diagnosis of a unicornuate uterus type A2 according to the American Fertility Society [8]. Amniocentesis at 16 gestational weeks was normal. Detailed scan at 20 gestational weeks showed a normal appearing fetus. The course of the pregnancy was uneventful until 34 weeks and 5 days when she had premature rupture of membranes. Due to nonreassuring fetal nonstress test, she had gone through a cesarean section. A baby girl of 2400 grams with APGAR scores of 8 and 10 was delivered.

**Figure 1:**
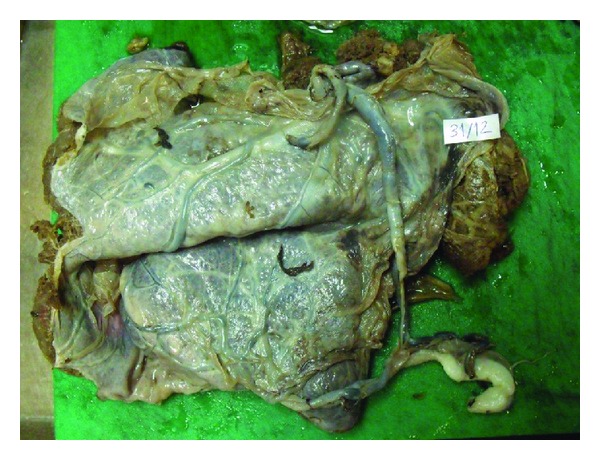
Velamentous and furcate cord insertion into the placenta.

The placenta did not expel spontaneously, so manual removal was performed. A wide portion of the placenta was hardly removed from the uterus but a small portion did not separate. A case of partial placenta accreta was diagnosed and this part was left attached to the uterus keeping the membraneous part out with the whole placenta. The placenta and the umbilical cord were sent to pathologic examination. To avoid postpartum hemorrhage Lynch compression sutures were performed. A single dose of methotrexate was given with intravenous antibiotics. Postpartum bleeding was normal. She was discharged on postpartum day 3 without any excessive bleeding and any sign of infection. Pathologic examination of the placenta and the umbilical cord revealed velamentous and furcate cord insertion (Figure 1).

One year after the delivery the patient did well, her menses were regular, and she is opted for another pregnancy. She was informed and counseled about the risks of placentation problems and the risk of another preterm delivery due to unicornuate uterus.

## 3. Discussion

We report a very rare case of velamentous and furcated cord insertion and placenta accreta in a woman with primary infertility and unicornuate uterus who achieved pregnancy on the fourth IVF attempt. Both velamentous and furcate cord insertions are serious obstetric conditions that could lead to cord rupture, bleeding, intrauterine fetal demise, and maternal death [3, 4]. Although there are some reports in the literature about the prenatal detection of velamentous cord insertion, it is not always possible to detect such insertion abnormalities [3].

Another important complication of our case is the partial placenta accreta. Instead of hysterectomy we chose to conservatively manage the patient to preserve her fertility [9]. The retained placental part left in situ and Lynch compression sutures were performed and a single dose of methotrexate was given.

The preterm delivery in this case can be tied to the unicornuate uterus which is a condition usually associated with this adverse event in the literature [7]. Also the poor reproductive outcome can also be related to this uterine anomaly [7].

As a result of this serious case of placental and umbilical cord abnormalities we may suggest that in an IVF pregnancy with uterine abnormality obstetricians should be cautious about the placenta and the umbilical cord and they should carefully scan the placenta and the cord insertions to avoid the risk of unanticipated operations.
